# High Glucose Accelerates Cell Proliferation and Increases the Secretion and mRNA Expression of Osteopontin in Human Pancreatic Duct Epithelial Cells

**DOI:** 10.3390/ijms18040807

**Published:** 2017-04-12

**Authors:** Miho Ito, Naohiko Makino, Akiko Matsuda, Yushi Ikeda, Yasuharu Kakizaki, Yoshihiko Saito, Yoshiyuki Ueno, Sumio Kawata

**Affiliations:** 1 Department of Gastroenterology, Faculty of Medicine, Yamagata University, Yamagata 990-9585, Japan; i-miho@ypch.gr.jp (M.I.); kokia7792@gmail.com (A.M.); ikeda_yushi@hotmail.com (Y.I.); y-kakizaki@med.id.yamagata-u.ac.jp (Y.K.); y-saitou@med.id.yamagata-u.ac.jp (Y.S.); y-ueno@med.id.yamagata-u.ac.jp (Y.U.); 2 Hyogo Prefectural Nishinomiya Hospital, Nishinomiya 662-0918, Japan; Sumio_Kawata@pref.hyogo.lg.jp

**Keywords:** human pancreatic duct epithelial cell line (HPDE-6), osteopontin, diabetes, hyperinsulinemia, pancreatic ductal adenocarcinoma

## Abstract

Background: The incidence of pancreatic cancer is increasing year-by-year in Japan. Among the diseases that complicate pancreatic cancer, diabetes is the most common. Recently, it has become evident that patients suffering from diabetes and obesity show increased expression of osteopontin (OPN). The purpose of this study was to investigate the effect of high glucose and high insulin culture conditions on a human pancreatic duct epithelial cell line (HPDE-6), focusing particularly on OPN expression. Methods: HPDE-6 were cultured under various conditions, employing several combinations of glucose (normal, 6 mM high, 30 mM, and 60 mM) and insulin (0.1 nM, 1 nM) concentration. Results: HPDE-6 cell proliferation was significantly accelerated under high glucose culture conditions in comparison to samples in 6 mM glucose, and was more prominent under high insulin conditions. At the same time, the expression of OPN mRNA was also increased significantly. In comparison with 6 mM glucose, the expression of 8-OHdG DNA was increased in high glucose culture. Conclusion: HPDE-6 cells show accelerated proliferation and increased OPN expression when cultured under high glucose and high insulin conditions. Furthermore, the cells show increased oxidative stress in the presence of high glucose.

## 1. Introduction

In clinical practice, it is known that many cases of pancreatic cancer become evident when patients are investigated for a worsening in glycemic control. Among diseases that are complications of pancreatic cancer, diabetes is the most common, with an incidence as high as 25.9% in the pancreatic cancer registry report of 2007 (Committee for Pancreatic Cancer Registry, Japan Pancreas Society) [1]. There have been many arguments regarding whether or not diabetes is the cause or the result of pancreatic cancer [2,3,4,5,6]; however, details of the underlying molecular biology remain obscure. Huxley et al. performed a meta-analysis of 9220 cases of pancreatic cancer in 36 reports covering a period from 1966 to 2005, and reported that the relative risk of pancreatic cancer in patients with diabetes was 1.82 (95% CI: 1.66–1.89) [4]. Moreover, based on 20 reports published between 1975 and 1994, Everhart and Wright performed a meta-analysis and concluded that the relative risk of pancreatic cancer in patients with diabetes was 2.1 (95% CI: 1.1–2.7) [5]. However, no previous reports have investigated the effect of hyperglycemia and hyperinsulinemia on pancreatic duct epithelium. In the present study, researchers focused on a cell line derived from the normal human pancreatic duct epithelium and examine the expression of osteopontin (OPN), a non-collagenous bone matrix phosphoprotein known to be present in many other cells, including epithelial cells, endothelial cells, and vascular smooth muscle cells. OPN shows accelerated expression in individuals with diabetes and obesity, and it is evident that OPN contributes to various diseases [7,8,9,10,11,12]. 

Several studies describe OPN as a critical regulator of insulin resistance and diabetes mellitus. OPN expression in adipose tissue as well as serum OPN levels were substantially elevated in obese patients compared with lean subjects, and were further increased in obese diabetic or insulin resistant patients [9,13]. OPN has been implicated widely in tumor growth and metastasis, particularly liver, lung, and breast [14,15,16]. The most appropriate serum biomarker for pancreatic cancer is CA19–9, but serum OPN is also used as a diagnostic biomarker for pancreatic cancer [17]. This study investigated the effect of high glucose and high insulin concentrations on a normal human pancreatic duct epithelial cell line, particularly focusing on the expression of OPN. 

## 2. Results

### 2.1. Cell Proliferation

**Figure 1:**
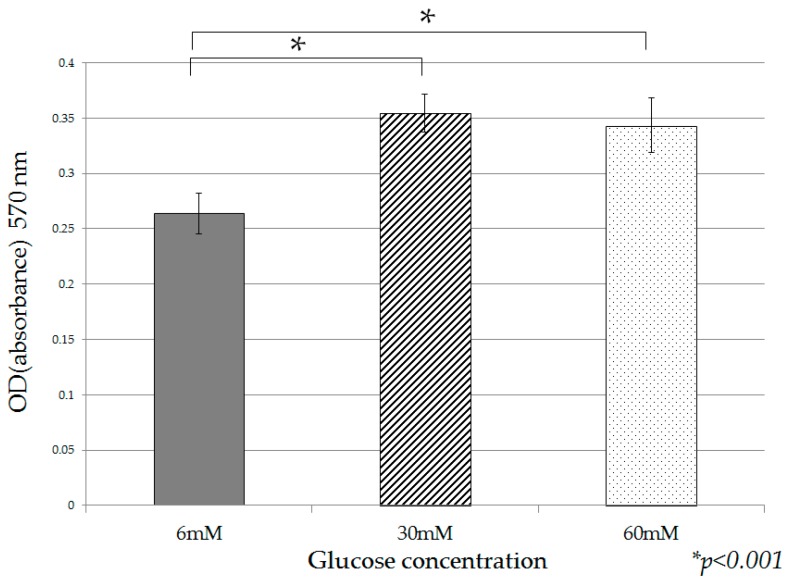
Human pancreatic duct epithelial cell line (HPDE-6) cell proliferation under different glucose culture conditions. After 120 h (Day 5) of culture under different glucose culture conditions, the cells were assessed for potential growth using the MTT assay. Data are mean ± SD. * *p* < 0.001 compared with normal glucose (6 mM). This experiment was repeated three times for reproducibility. Data were analyzed using Dunnett’s multivariate comparison.

**Figure 2:**
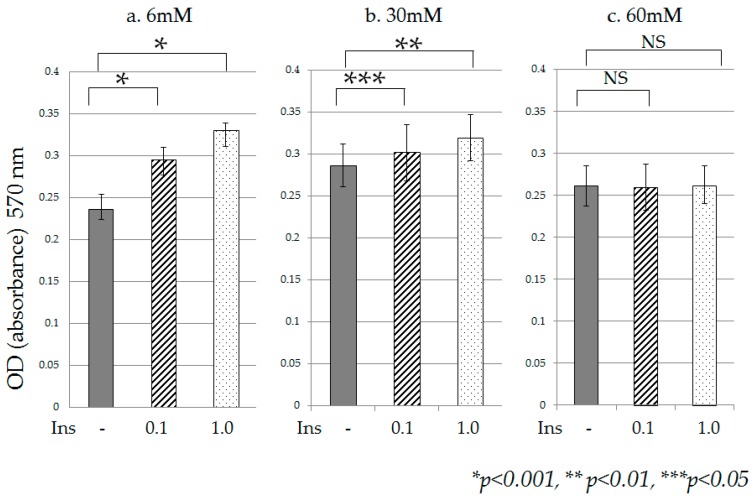
Proliferation of HPDE-6 cells under high-insulin conditions. After 120 h of culture under different glucose ((**a**) 6 mM, (**b**) 30 mM glucose, (**c**) and 60 mM glucose), and insulin culture conditions (no addition, 0.1 nM, and 1 nM), the cells were assessed for potential growth using the MTT assay. The horizontal line shows insulin conditions (nM). Accelerated cell proliferation was observed when high-insulin conditions (0.1 nM, 1 nM) were added to cultures containing 6 mM glucose, and 30 mM glucose. However, in the presence of 60 mM glucose, no change in cell proliferation due to high insulin was observed. Data are mean ± SD. * *p* < 0.001 compared with no addition insulin. ** *p* < 0.01 compared with no addition insulin *** *p* < 0.05 compared with no additional insulin. NS: non-significant. Each experiment was repeated three times for reproducibility. Data were analyzed using Dunnett’s multivariate comparison.

Under high glucose culture conditions (30 mM, 60 mM), cell proliferation was accelerated on Day 2 in comparison with normal glucose culture conditions (6 mM). Significant acceleration of cell proliferation was observed in the presence of 30 mM and 60 mM glucose on Day 5, in comparison with 6 mM (Figure 1). Furthermore, accelerated cell proliferation was observed when high-insulin conditions (0.1 nM, 1 nM) were added to cultures containing 6 mM glucose (Figure 2a), and 30 mM glucose (Figure 2b). However, in the presence of 60 mM glucose, no change in cell proliferation due to high insulin was observed (Figure 2c).

### 2.2. Quantification of OPN mRNA

**Figure 3:**
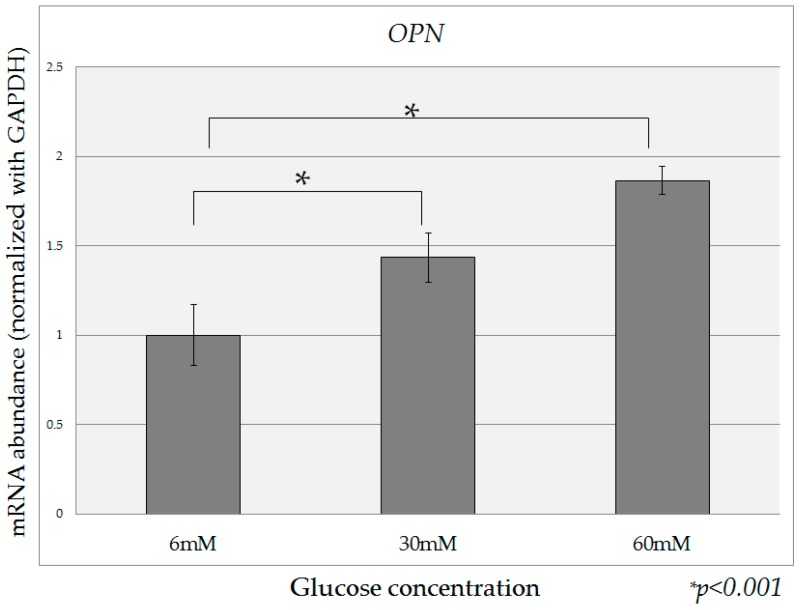
Quantification of *OPN* mRNA. Compared to the expression of *OPN* mRNA in HPDE-6 cells cultured in the presence of 6 mM glucose, the expression of *OPN* mRNA in the presence of high glucose (30 mM, 60 mM) was significantly increased. Data are mean ± SD. * *p* < 0.001 compared with normal glucose (6 mM). This experiment was repeated three times for reproducibility. Data were analyzed using Dunnett’s multivariate comparison.

**Figure 4:**
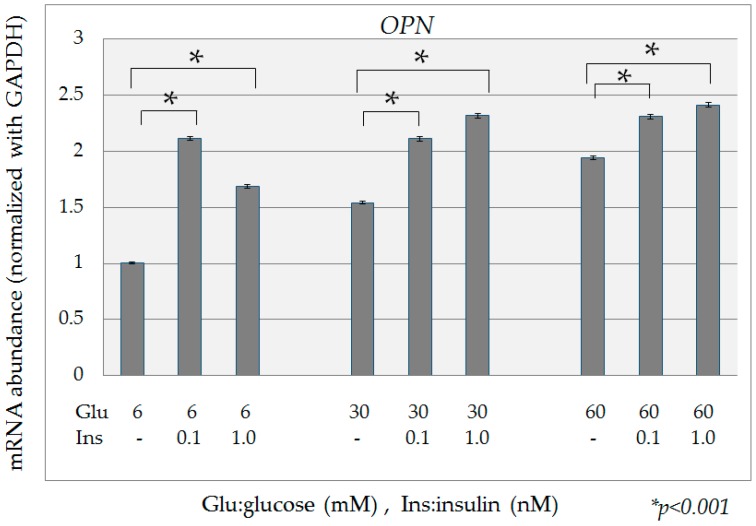
Quantification of *OPN* mRNA with high-insulin conditions. When high-insulin conditions (0.1 nM, 1 nM) were added to various concentrations of glucose in culture, expression of *OPN* mRNA was significantly increased in all cases relative to that in the absence of insulin. Data are mean ± SD. * *p* < 0.001 compared with normal glucose (6 mM). This experiment was repeated 3 times for reproducibility. Data were analyzed using Dunnett’s multivariate comparison.

Compared to the expression of *OPN* mRNA in HPDE-6 cells cultured in the presence of 6 mM glucose, expression of *OPN* mRNA in the presence of high glucose (30 mM, 60 mM) was significantly increased (Figure 3). When high-insulin conditions (0.1 nM, 1 nM) were added to various concentrations of glucose in culture, the expression of *OPN* mRNA was significantly increased in all cases relative to that in the absence of insulin (Figure 4).

### 2.3. OPN Protein Expression Analysis

**Figure 5:**
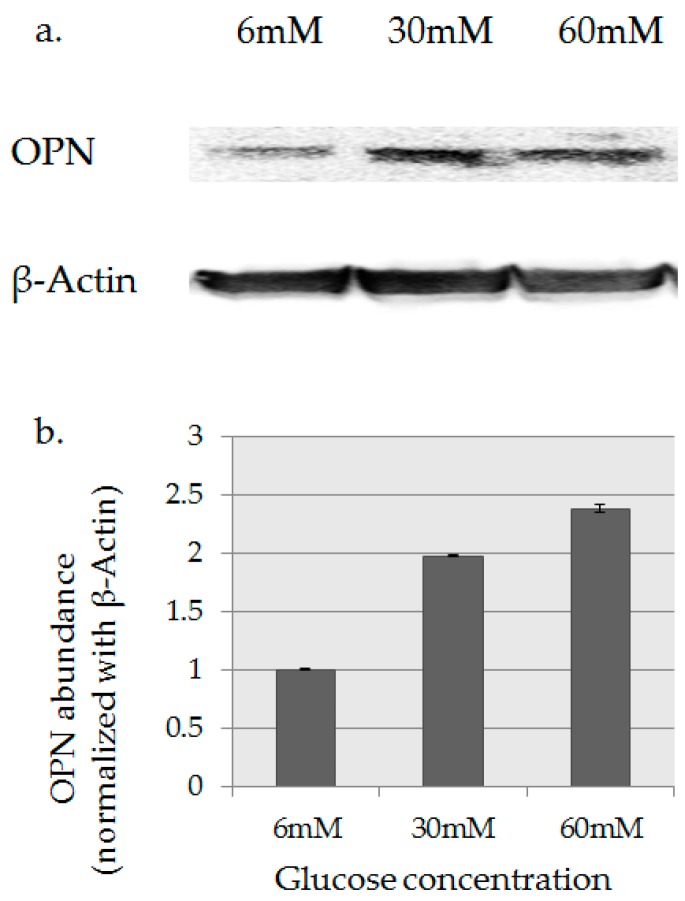
OPN protein expression analysis. (**a**) Western blotting. OPN protein expression in high-glucose culture (30 mM, 60 mM) was higher than in the presence of 6 mM glucose. (**b**) The integrated luminance value (OPN/β-actin) of the band extracted using the Image Analysis Software.

The expression of OPN protein in HPDE-6 cells was confirmed by Western blotting. A Comparison of bands detected used an AE-9150 Ez-Capture II (Atto Corp., Tokyo, Japan). OPN protein expression in high-glucose culture (30 mM, 60 mM) was higher than in the presence of 6 mM glucose (Figure 5a). The integrated luminance value (OPN/β-actin) of the band extracted using the Image Analysis Software CS Analyzer (Atto Corp., Tokyo, Japan) is shown in Figure 5b.

### 2.4. OPN Gene Inhibition Using OPN-siRNA

In the experiment using siRNA, the effect of OPN inhibition on the growth potential of HPDE-6 was investigated in the presence of 6 mM glucose (normal) and high glucose (30 mM). After 48 h, the inhibition effect of OPN was investigated by real time PCR.

**Figure 6:**
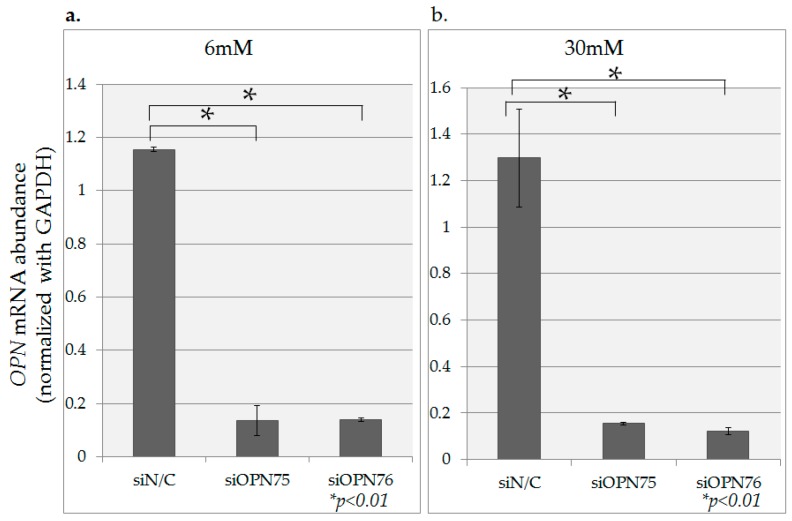
The Inhibitory effect of *OPN* expression by OPN-siRNA. *OPN* gene inhibition due to transfection with two types of OPN-siRNA (siOPN75, siOPN76) was confirmed in 6 mM glucose and 30 mM glucose condition. Data are mean ± SD. * *p* < 0.01 compared with negative control (siN/C). This experiment was repeated twice for reproducibility. Data were analyzed using Dunnet’s multivariate comparison.

**Figure 7:**
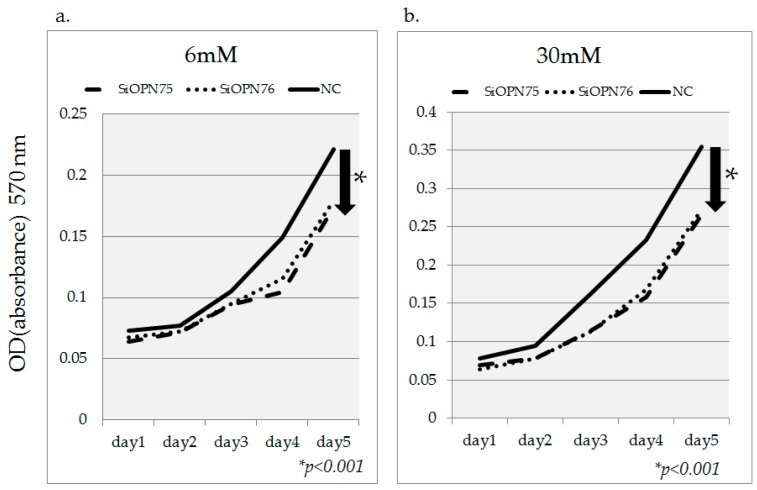
The effects of *OPN* gene inhibition using OPN-siRNA toward cell proliferation. *OPN* gene inhibition due to transfection with two types of OPN-siRNA (siOPN75, siOPN76) was confirmed in 6 mM glucose and 30 mM glucose condition. Down arrows in the figure indicate that concomitant inhibition of cell proliferation was observed significantly at day 5. Data are mean ± SD. * *p* < 0.001 compared with negative control (NC). This experiment was repeated twice for reproducibility. Data were analyzed using Student’s *t*-test.

*OPN* gene inhibition due to transfection with two types of OPN-siRNA (siOPN75, siOPN76) was confirmed in the presence of both 6 mM and 30 mM glucose (Figure 6). Concomitant inhibition of cell proliferation was also observed under both conditions. The cells were assessed for potential growth using the MTT assay under 6 mM and 30 mM glucose culture conditions as shown in Figure 7.

### 2.5. 8-OHdG Detection

**Figure 8:**
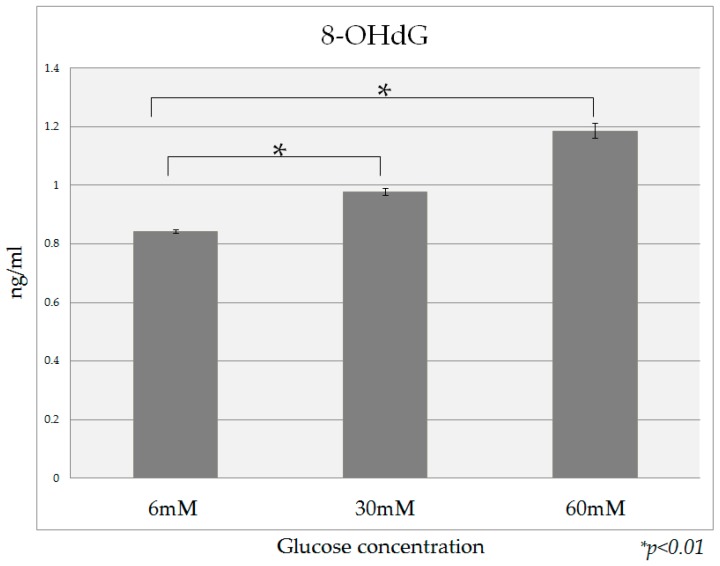
The dose dependent effects of glucose on 8-OHdG detection. The 8-OHdG content of DNA in HPDE-6 cells cultured in the presence of 30 mM and 60 mM glucose was significantly higher than that in the presence of 6 mM glucose. Data are mean ± SD. * *p* < 0.01 compared with normal glucose (6 mM). This experiment was repeated 3 times for reproducibility. Data were analyzed using Dunnett’s multivariate comparison.

The 8-OHdG content of DNA in HPDE-6 cells cultured in the presence of 30 mM and 60 mM glucose was significantly higher than that in the presence of 6 mM glucose (Figure 8). When high-insulin conditions (0.1 nM, 1 nM) were added to all glucose culture conditions, no definite increase in the 8-OHdG content was observed. 

## 3. Discussion

Diabetes is the most common complication of pancreatic cancer, and a risk factor for it [1,2,3,4,5,6]; however, the molecular biologic mechanism involved has not yet been clarified. Early detection of pancreatic cancer is necessary for an improved prognosis, and delineation of patients who are at high risk is a major issue. Clarifying the pathophysiology of diabetes in the pancreatic duct epithelium is believed to be very important for achieving the goal of early detection of pancreatic cancer. In this experiment, an accelerated proliferation of HPDE-6 cells was observed under high glucose and high insulin culture conditions. Very few studies have investigated the relationship between proliferation of the pancreatic duct epithelium and the carcinogenesis of pancreatic cancer [18,19,20]. Using Ki67 immunostaining of autopsy pancreas tissue, Butler et al. found that proliferation of the pancreatic duct epithelium was accelerated in diabetic and obese patients [18], thus corresponding to the results of the present study. Accordingly, hyperglycemia due to diabetes is involved in accelerated proliferation of the pancreatic duct epithelium, and hyperinsulinemia, observed in obese patients with insulin resistance, also plays a role in this respect. However, we should have added at least one other method to show cell proliferation; this is one of the limitations of our study.

Li et al. compared and investigated the treatment regimens used for diabetes in relation to the rate of pancreatic carcinogenesis, and reported that while insulin analogs and insulin secretagogues increased the risk of pancreatic cancer onset in diabetic patients by approximately 4.99-fold and 2.52-fold, respectively. Metformin, which is a drug used for treatment of insulin resistance, but does not increase the insulin concentration in blood, reduced the risk of pancreatic cancer by 62%, particularly when metformin treatment was continued for five years or longer [21]. Metformin is known to have a direct effect on the activation of AMP-activated protein kinase (AMPK), and affects cell proliferation and apoptosis via p53 and p27kip1. Furthermore, protein synthesis and cell growth are inhibited due to inhibition of the mammalian target of rapamycin (hereinafter, mTOR) [22]. Yang et al. reported that the molecular mechanism involved in cell proliferation via AMPK and mTOR is involved in pancreatic carcinogenesis with a background of diabetes [23]. These reports indicate that various molecular mechanisms are involved in the cell proliferation response to hyperglycemia and hyperinsulinemia, and that clarifying these mechanisms might lead to future prevention and treatment of pancreatic cancer.

This study suggests that OPN may be involved in cell proliferation. Although the mechanism of action of OPN in the pancreatic duct epithelium is not clear, some reports have mentioned that hyperglycemia is involved in the proliferation of vascular smooth muscle cells (VSMC) via up-regulation of OPN [24], and it is believed that increased OPN expression is due to hyperglycemia and hyperinsulinemia in the pancreatic duct epithelium, which in turn accelerates cell proliferation. OPN has long been known to be involved in diabetes and obesity [7,8,9,10]. Takemoto reported in 1999 that OPN expression is accelerated in the vascular smooth muscle cells of rats due to high glucose via activation of protein kinase C and the hexosamine pathway [7] Moreover, Hsieh et al. reported that oxidative stress is involved in the accelerated OPN expression in vascular smooth muscle cells of rats due to high glucose [8]. Furthermore, OPN is implicated in the progression and metastasis of multiple cancers [14,15,16]. Kothari et al. reported that the epithelial-mesenchymal transition (EMT) is being recognized as a significant contributor to tumor progression, OPN is able to guide EMT through specific cellular signaling pathways and by restructuring the microenvironment to modify EMT programs [25]. Chipitsyna et al. suggest that nicotine may contribute to pancreatic cancer pathogenesis through up-regulation of OPN [26]. In our experiment, researchers were not able to clarify how high glucose and high insulin are involved in increased OPN expression in HPDE-6, how OPN is involved in the cell proliferation of OPN, or the mechanism or pathway thereof. However, OPN, which is attracting attention for its involvement with diabetes and obesity in multiple organs, is surmised to play a major role in the pancreas under hyperglycemia and high-insulin conditions as well, and it is believed that the action mechanism requires clarification.

In this study, 8-OHdG, which is a marker of DNA oxidative stress, was more highly expressed under high glucose culture conditions than in the presence of a normal glucose concentration, suggesting that HPDE-6 cells cultured under high glucose conditions are exposed to a higher degree of oxidative stress. To investigate the mechanism of oxidative stress generation in diabetics, Giardino et al. cultured vascular endothelial cells in the presence of a high sugar concentration and found that although reactive oxygen species (ROS) did not increase in the culture medium, increased oxidative stress due to diabetes occurred in the cells [27]. Nishikawa et al. investigated the involvement of the mitochondrial electron transport system as the source for intracellular ROS production in diabetes, and reported that the generation of mitochondria mediated ROS played a major role in the expression of intracellular metabolic disorder due to high glucose [28]. Moreover, it has been reported that the ROS generated in this manner damage the genomic DNA involved in cell proliferation in various ways, and may be involved in carcinogenesis [29]. Specifically, 8-OHdG generated on the chromosome DNA induces from G to T mutation during DNA replication, so it is believed that an increase of 8-OHdG in chromosomes is related to an increased risk of carcinogenesis [30]. It is believed that hyperglycemia creates oxidative stress, which can damage DNA in the pancreatic duct epithelium through the same mechanism, leading to the onset of pancreatic cancer. We have not research how antioxidant treatment will affect the 8-OHdG and OPN expression level in HPDE-6 cells. Therefore, measurement of ROS in high glucose treated cells and comparing to control cells, or inhibition of ROS to see if it reverses 8-OhdG is needed to support the conclusion of increased oxidative stress in high glucose condition and ROS induced 8-OHdG expression, we would like to consider this in further research.

Existing data indicates that hyperglycemia and hyperinsulinemia are involved in the accelerated proliferation of pancreatic duct epithelial cell. Moreover, it is suggested that OPN may be involved in the proliferation of pancreatic duct epithelial cells. However, the present study investigated only a single cell line derived from normal human pancreatic duct epithelium, and it seems to be feasible to investigate other cell lines to confirm these results.

**Figure 9:**
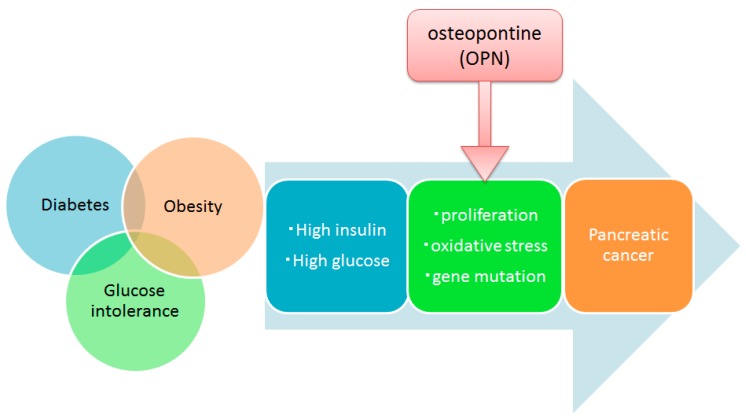
Possible mechanism of carcinogenesis in obesity and diabetes. Hyperglycemia and hyperinsulinemia due to diabetes, obesity and glucose intolerance are believed to be involved in the accelerated proliferation of pancreatic duct epithelial cells. Various molecular mechanisms are involved in carcinogenesis due to hyperglycemia and hyperinsulinemia, and clarification of these mechanisms will lead to methods for prevention and treatment of pancreatic cancer.

Hyperglycemia and hyperinsulinemia due to diabetes, obesity and glucose intolerance may be involved in accelerated cell proliferation in the pancreatic duct epithelium cells. Various molecular mechanisms including OPN are involved in carcinogenesis due to hyperglycemia and hyperinsulinemia, and clarification of these mechanisms will lead to methods for prevention and treatment of pancreatic cancer (Figure 9). 

## 4. Materials and Methods

### 4.1. Cell Culture

HPDE-6, a cell strain derived from normal human pancreatic duct epithelial cells, was used for the study at passages 28–32. The cells were provided by Professor T. Furukawa, Tokyo Women’s Medical University [31]. The medium employed was a mixture of HKGS (Thermo Fisher Scientific K.K., Waltham, MA, USA) and Medium 154S (Thermo Fisher Scientific K.K.). In all experiments, HPDE-6 cells were cultured in an incubator at 37 °C in the presence of 5% CO_2_. 

### 4.2. Evaluation of Cell Proliferation 

HPDE-6 cells were inoculated into a 96-well cell culture plate at 5000 cells per well (0.32 cm^2^), and then transferred to medium with different glucose and insulin concentrations 24 h later. After another 24 h (Day 1) until 120 h (Day 5), the cells were assessed for potential growth every 24 h using the MTT (3-(4,5-dimethylthiazol-2-yl)-2,5-diphenyltetrazolium bromide (Thermo Fisher Scientific K.K.)) assay. Three different glucose concentrations were employed: 6 mM (108 mg/dL), 30 mM, and 60 mM, the lowest regarded as normal. Three different insulin conditions were also employed, to give a total of nine combinations with glucose: No addition, 0.1 nM, and 1 nM. When changing the glucose and insulin concentrations, d-glucose (Thermo Fisher Scientific K.K.) and insulin (OriGene EU Acris Antibodies GmbH, Herford, Germany)) were added to the normal medium and adjusted. 

### 4.3. RNA/DNA/Protein Extraction

HPDE-6 cells were inoculated into 100-mm dishes (BD Falcon, Tokyo, Japan) at 1 × 10^6^ cells/dish. The cells were transferred to each of the nine different media at 80% confluency in the same manner as when evaluating cell proliferation, and the cells were collected after 24 h. The AllPrep DNA/RNA/Protein Mini Kit (QIAGEN, Hilden, Germany) was used for extraction of RNA, DNA, and protein. The quantity and purity of RNA and DNA was determined using a NanoVue Plus micro-sample spectrum photometer (GE Healthcare, Little Chalfont, Bucks, UK).

### 4.4. Real-Time PCR

The levels of expression of messenger RNA (mRNA) for OPN, and glyceraldehyde 3-phosphate dehydrogenase (GAPDH) were analyzed. The extracted total RNA was reverse-transcribed to complementary DNA (cDNA) using a SuperScript VILO cDNA synthesis kit (Thermo Fisher Scientific K.K). Subsequently, a reaction mixture (20 μL) was made containing cDNA (100 ng/2 μL), 10 μL TaqMan Universal Master Mix II (Thermo Fisher Scientific K.K.), 1 μL TaqMan probe, and 7 μL nuclease-free water and this was used for measurement. OPN (ID: Hs00959010), and GAPDH (ID: Hs99999905) were selected as the TaqMan probes used for mRNA quantification and were supplied with the TaqMan Gene Expression Assays (Thermo Fisher Scientific K.K.). A 7500 Fast Real-Time PCR System (Thermo Fisher Scientific K.K.) was used according to the manufacturer’s protocol. The gene expression levels of OPN were expressed as a corrected value obtained by dividing the mRNA expression level by that for GAPDH. 

### 4.5. Protein Detection 

SDS-polyacrylamide gel electrophoresis (SDS-PAGE) was carried out using the protein obtained as described in Section 3, and the resulting gel was transcribed to an Immun-Blot PVDF membrane (Bio-Rad Laboratories, Inc., Hercules, CA, USA). OPN-b (X-25) (Santa Cruz Biotechnology, Inc., Heidelberg, Germany) was selected as the primary antibody and goat anti-rabbit IgG HRP (Santa Cruz Biotechnology, Inc. was selected as the second antibody for detection of OPN; Monoclonal Anti-β-Actin Clone AC-15 (Sigma-Aldrich, St. Louis, MO, USA) was used on the first antibody while Anti-Mouse IgG (Santa Cruz Biotechnology, Inc.) was used as the second antibody. This was extracted using AE-9150 Ez-Capture II (Atto Crop.), and the integrated luminance value of the extracted band was calculated using a CS Analyzer 3 (Atto Crop.). The expression of OPN protein was expressed as a corrected value obtained by dividing the integrated value of the bands under each condition by the integrated value of the band for β-actin. 

### 4.6. Gene Inhibition Experiment Using OPN-Small Interfering RNA (siRNA)

All siRNAs and reagents used for the gene inhibition experiment were purchased from Thermo Fisher Scientific K.K., and the siPORT NeoFX Transfection Agent was employed. siRNA75 (ID: s13375) and siRNA76 (ID: s13376) were used as the OPN-siRNA. Ambion’s Silencer Select Negative Control #1 siRNA (arrangement not disclosed) was used as the negative control. A gene inhibition experiment was carried out according to the product protocol. In the experiment using siRNA, the effect of OPN inhibition on the growth potential of HPDE-6 was investigated in the presence of 6 mM glucose (normal) and high glucose (30 mM). Subsequently, the MTT assay was performed every 24 h from 24 h (Day 1) until 120 h (Day 5). Moreover, after 48 h, the inhibition effect of OPN was investigated by real time PCR. For real-time PCR, cDNA was synthesized using a Cells-to-cDNA II Kit (Thermo Fisher Scientific K.K.) and the same OPN and Taqman probe for GAPDH as mentioned in Section 4 were used. 

### 4.7. Detection of 8-Hydroxydeoxyguanosine (8-OHdG) in Different Concentrations of Glucose

Each sample was hydrolyzed using an 8-OHdG Assay Preparation Reagent Set (Wako Pure Chemical Industries, Ltd., Tokyo, Japan) employing the DNA obtained as described in the method of RNA/DNA/protein extraction. Subsequently, 8-OHdG was detected using High Sensitive 8-OHdG Check (Wako Pure Chemical Industries, Ltd.) in accordance with the product protocol.

### 4.8. Statistical Analysis 

All results are expressed as the mean value ± standard deviation. The SAS (Statistical Analysis System) software package was used for statistical analysis. A *t*-test was used for comparisons between two groups, and Dunnett’s multivariate comparison was used for analysis of three or more groups after primary arrangement variance analysis. Differences at *p* < 0.05 were considered statistically significant.

## 5. Conclusions

HPDE-6 cells show accelerated proliferation and increased OPN expression when cultured under high glucose and high insulin conditions. Furthermore, the cells show increased oxidative stress in the presence of high glucose.
